# The effect of a social network-based intervention to promote HIV testing and linkage to HIV services among fishermen in Kenya: a cluster-randomised trial

**DOI:** 10.1016/S2214-109X(24)00539-4

**Published:** 2025-04

**Authors:** Carol S Camlin, Lila A Sheira, Zachary A Kwena, Edwin D Charlebois, Kawango Agot, James Moody, Benard Ayieko, Sarah A Gutin, Antony Ochung, Phoebe Olugo, Jayne Lewis-Kulzer, Holly Nishimura, Monica Gandhi, Elizabeth A Bukusi, Harsha Thirumurthy

**Affiliations:** Department of Obstetrics, Gynecology & Reproductive Sciences (Prof C S Camlin PhD, L A Sheira PhD, J Lewis-Kulzer MPH), Department of Medicine (Prof C S Camlin, Prof E D Charlebois PhD, S A Gutin PhD, H Nishimura PhD, Prof M Gandhi MD), School of Medicine, University of California, San Francisco, CA, USA; Kenya Medical Research Institute, Kisumu, Kenya (Z A Kwena PhD, E A Bukusi MBChB); Impact Research & Development Organization, Kisumu, Kenya (K Agot PhD*, B Ayieko MBChB, A Ochung BS, P Olugo BS); Department of Sociology, Duke University, Durham, NC, USA (Prof J Moody PhD); Department of Community Health Systems, School of Nursing, University of California, San Francisco, CA, USA (S A Gutin); Department of Medical Ethics and Health Policy, Perelman School of Medicine, University of Pennsylvania, Philadelphia, PA, USA (Prof H Thirumurthy PhD)

## Abstract

**Background:**

In sub-Saharan Africa, highly mobile men such as fishermen have a low uptake of HIV testing, prevention, and treatment. This study aimed to examine whether a HIV status-neutral, social network-based intervention could improve testing and linkage to prevention and treatment among fishermen in Kenya.

**Methods:**

The *Owete* cluster-randomised trial mapped the male social networks of fishermen in three communities along Lake Victoria in Siaya County, Kenya, and identified distinct social networks (clusters) with a highly connected, network-central man (promoter) in each network. Participant inclusion criteria were age 18 years or older, a listing in the Beach Management Unit registry, a governmental requirement to work as a fisherman at each site, and no participation in any other HIV-related study. Clusters were randomly assigned to an intervention group in which promoters were trained and offered multiple HIV self-tests to offer to cluster members, and transport vouchers (US$4) to encourage those members to link to HIV treatment or pre-exposure prophylaxis (PrEP). In control clusters, promoters received HIV information and referral vouchers for a free self-test or provider-administered test in nearby clinics that they were encouraged to offer to cluster members. We compared self-reported HIV testing in the past 3 months and linkage to HIV services among participants in intervention and control clusters at the 3-month follow-up visit in the intention-to-treat sample, defined as members of the social networks who were successfully contacted at 3 months using a cluster-adjusted two-sample test of proportions. The trial is registered at ClinicalTrials.gov, NCT04772469, and is completed.

**Findings:**

Between July 17, 2020 and Feb 2, 2022, 1509 eligible men participated in the beach census. 575 were excluded due to not being mapped to a close social network, and 934 men in 156 social network clusters were mapped. 453 men were randomly assigned to the intervention group and 481 were randomly assigned to the control group. 733 men completed a baseline survey (374 in the intervention group and 359 in the control group). 353 men in the intervention group and 313 in the control group completed the 3-month follow-up assessments and were included in the analysis of the primary outcome. Participants’ median age was 35·5 years (IQR 30·1–42·3); 85% were married, with 22% in polygynous relationships. HIV testing via any modality at 3 months was higher in intervention compared with control clusters (65·6 [95% CI 59·5–71·7] *vs* 31·3% [25·4–37·2], p<0·0001). Self-reported HIV testing at 3 months was also higher in intervention clusters (60·4% [95% CI 54·2–66·7] *vs* 10·0% [6·8–13·3], p<0·0001). Additionally, following testing, linkage to HIV treatment or PrEP among those who tested was higher in intervention clusters (67·3% [95% CI 61·2–73·5] *vs* 15·6% [10·9–20·2], p<0·0001).

**Interpretation:**

A status-neutral social network intervention that utilised HIV self-screening tests proved to be highly effective in engaging hard-to-reach, highly mobile Kenyan men in HIV testing and care. This strategy holds promise for improving early detection and care engagement for other infectious and non-communicable diseases globally. Similar approaches that leverage peer influence within social networks and the growing accessibility of self-screening tests could be adapted for conditions such as tuberculosis, hepatitis, and hypertension in diverse global health settings.

## Introduction

The engagement of men in HIV testing, prevention, and treatment remains suboptimal in many countries in sub-Saharan Africa. Despite great progress, the target of more than 95% of people living with HIV knowing their HIV status has not been fully met in the region, in part because men are less likely than women to test for HIV.^1,2^

They are also less likely to engage in HIV treatment and pre-exposure prophylaxis (PrEP),^1–3^ and more likely to default from treatment and have virological failure on antiretroviral therapy (ART).^3,4^ This results in men’s lower life expectancy on ART (relative to women’s),^2,4,5^ as well as their continued onward transmission of HIV to women.

In east Africa, a high-priority population of men are those working in the inland fishery settings at the Lake Victoria basin. The burden of HIV has historically been very high in Kenya’s fishing communities and remains so: recent studies estimate HIV incidence to be 4·6–6·9 per 100 person-years,^6^ with HIV prevalence of 9·5–19·1%,^7^ yet treatment coverage is only 53–61%.^8^ One of the contributors to high HIV risk is the mobility of fisherfolk, as fishermen often travel across the lake to follow the fish and female traders follow fishermen and circulate between beaches and regional markets.^9^ Another contributing factor is a transactional sex economy embedded within the fish trade.^9,10^ The low rates of HIV testing and care engagement among fishermen are also influenced by structural factors related to their work; fishermen have difficulty accessing services at fixed-location clinics and during typical clinic hours. Cultural and behavioural factors that might influence lower engagement in testing and services include HIV-related stigma and gender norms that counter men’s health-seeking behaviours (eg, men engaging in so-called proxy HIV testing, assuming their own HIV status based on their female partners’ test results).^11,12^

To address these gaps, the *Owete* (“brothers” in Dholuo) study aimed to determine whether an HIV status-neutral, social network-based approach could improve HIV testing as well as linkage to ART treatment and prevention (daily oral PrEP) among fishermen in Kenya. To promote testing, we relied on HIV self-testing, which can overcome barriers to clinic-based, provider-administered testing. Although several studies in sub-Saharan Africa have found that secondary distribution of self-tests by women to their male partners is an effective way to promote partner and couples testing,^13–15^ no previous studies have rigorously evaluated the effect of secondary distribution of self-tests from men to their male friends on HIV testing and linkage to care. We sought to leverage the power of social influence by selecting highly socially connected men within social networks to serve as promoters, who would not only distribute self-tests to men in their networks but also motivate those men to link to health facilities for confirmatory testing and for ART or PrEP. The intervention also included low-cost financial incentives, which have been shown to further increase linkage to treatment and prevention.^16^

## Methods

### Study design and participants

**Figure 1:**
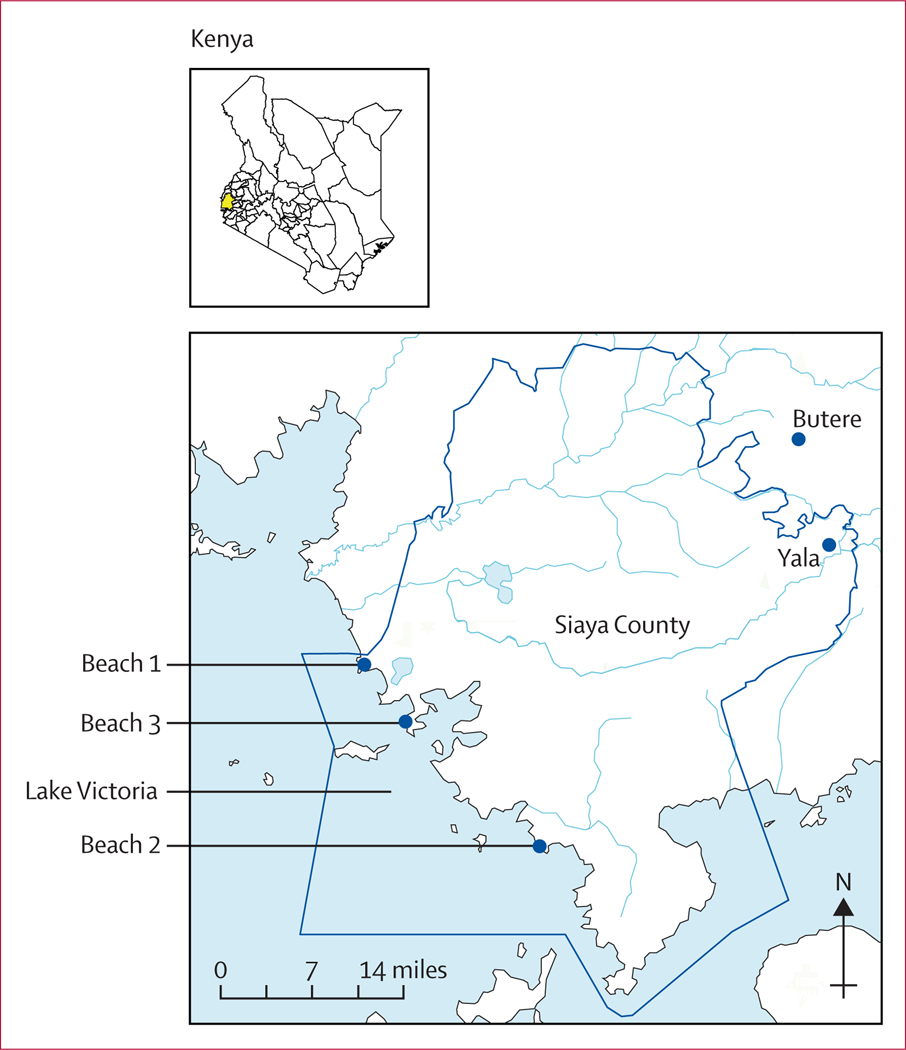
Map of study communities in Siaya County, Kenya

The *Owete* study is a cluster-randomised controlled trial which used a social network-based approach for the distribution of HIV self-tests to fishermen working in three communities along Lake Victoria in Siaya County, Kenya (figure 1). Study methods have been previously described^17^ and we provide a brief overview here. Within each beach community, a complete census of men’s social networks and subsequent mapping of social network structures was conducted among men who met the following eligibility criteria: (1) aged 18 years or older; (2) listed in the Beach Management Unit registry, a governmental requirement to work as a fisherman at each site; and (3) not participating in any other HIV-related study. This study was approved by the Kenya Medical Research Institute Scientific Ethics Review Unit (KEMRI-SERU, 677), National Commission for Science Technology (NACOSTI/P/23/24591), and the University of California, San Francisco Institutional Review Board (UCSF-IRB, 19–28205). All participants gave written informed consent. Study oversight was provided by an independent data and safety monitoring board, which reviewed reports of all potential safety events. The study was registered at ClinicalTrials.gov, NCT04772469, and is completed.

### Randomisation and masking

Within each beach, social network clusters were randomised (1:1) by alternating assignment of randomly chosen leaders (using uniform random sampling from a risk-set defined by having high centrality and matched 1:1 by degree to control cases) and their clusters to either receive the intervention (leaders were invited to serve as distributors of HIV self-tests to members of their social networks along with monetary vouchers to incentivise their post-test linkage to services) or control (leaders were invited to distribute referral vouchers to their network members that encouraged clinic-based HIV testing or free HIV self-test pickup at those clinics). Allocation status was conducted by JM and was masked to the study team and participants until after baseline was conducted, at which point network-central leaders for each cluster were invited to be promoters for the intervention and control group components.

### Procedures

We administered a baseline survey to men who met the eligibility criteria, were successfully reached, and provided informed consent. This included men who were selected as network-central leaders. The baseline survey assessed participant demographic and socioeconomic characteristics, HIV testing history and engagement with prevention or care, and self-reported sexual behaviour. No ethnicity data were collected. We also contacted all participants at 3 months and administered a follow-up survey, which included follow-up questions on socio-economic and demographic characteristics, sexual behaviour, and HIV testing and linkage behaviour in the past 3 months. Additionally, the survey assessed interactions with the network-central leaders, and among leaders assessed their experiences with promoting HIV testing and linkage with men in their networks, and with distributing HIV self-test kits (among intervention arm leaders).

In intervention clusters, the leader of each social network was given oral fluid-based HIV self-tests to offer to the network members as well as monetary vouchers that incentivised the network members to seek clinic-based confirmatory testing after self-testing. Specifically, they were invited to a 2-day training on basic HIV and AIDS literacy, how to use self-tests, and how to promote referral to clinics for confirmatory testing and receipt of PrEP or ART. These promoters were told which men were deemed to be part of their mapped social network, given the number of tests sufficient for the size of their network, and encouraged to distribute the tests to the men in their network. Although the intervention did not track the recipients of the self-test distribution, study team members reminded and encouraged the network-central promoters to distribute the self-tests to their network members. All self-tests distributed to network- central promoters in the intervention group clusters were affixed with information for a study-affiliated clinic and information regarding the small monetary voucher for travel (500 Kenyan Shillings or ~US$4 during the study period), available upon linkage to the clinic independent of their test result. In control clusters, the leader of each social network was asked to distribute referral vouchers to their network members with information for a study-affiliated clinic, and to encourage members of their network to seek HIV testing and linkage to care or prevention services. Network-central promoters in control clusters were trained on basic HIV and AIDS literacy and how to promote referrals to local clinics to get tested. They were given vouchers with no monetary compensation that were redeemable for routine HIV testing at nearby clinics or a free self-test that could be picked up at nearby clinics.

### Outcomes

The primary outcomes were: (1) self-reported HIV testing within 3 months of the promoters being provided with self-tests (intervention) or vouchers for routine HIV testing or clinic-based self-test pickup (control), assessed in the follow-up survey; and (2) linkage to a study-affiliated health-care facility. HIV testing was defined in two ways: testing via blood or self-test and testing with a self-test only. We also report the following secondary outcomes: (1) ART use among those whose confirmatory test was positive, or (2) PrEP screening and initiation among those whose confirmatory test was negative and who were not already taking PrEP (per clinic records). Outcomes were assessed in the intention-to-treat sample, defined as members of the social networks who were successfully contacted at 3 months The remaining preregistered primary outcomes (viral suppression and adherence to PrEP) are reported elsewhere.

### Statistical analysis

The sample size calculations have been published elsewhere;^17^ in summary, assuming a minimum proportion of 38% in the control group and 52% in the intervention group, an intra-class correlation coefficient (ICC) of 0·15, and 80 clusters randomly assigned 1:1 to either the intervention or control group with an average size of eight, and a two-sided alpha of 0·05, the study had 80% power to detect a difference in HIV testing between groups. Input parameters, such as the estimated proportion of participants who would use a self-test and the ICC, were informed from the SEARCH study and previous HIV self-testing studies that relied on secondary distribution of self-tests.^13,17^ No interim analyses were conducted, and no stopping guidelines were established.

We generated summary statistics among the mapped fishermen sample with whom we conducted a baseline survey. We defined our intention-to-treat sample as those who were randomly assigned to intervention and control groups (regardless of whether they received a self-test or voucher from their promoter). We compared self-reported HIV testing at the 3-month follow-up visit by group among the intention-to-treat sample using a two-sample test of proportions with an additional control for cluster by including the ICC and cluster variable as specifications. We then conducted an intention-to-treat multivariable logistic regression analysis using two-level models that controlled for the beach community as the stratification factor in the randomisation and included cluster-robust standard errors. Participants with missing data were coded as failure to test or link unless known to be living with HIV (per health facility records) or known to have died before the end of the evaluation period. No subgroup nor adjusted analyses were conducted. All statistical analyses were conducted using Stata 16, network analysis and randomisation used SAS 9.4, network visualisations were generated in Pajek (version 5.17), and data management systems and visualisations were developed in R (version 4.0.5).

Social network data were collected as part of the Beach Management Unit registry census completed before the baseline survey. The social network surveys ascertained up to seven types of relations among fishermen using a name-generator. Participants were asked to name the men in their community to whom they were most closely connected, for relation types including health (whom they would seek health information from or share information with), finances (to whom they would turn to borrow money), and spending time (on work and social activities). We then used the social network data and a name matching algorithm to identify “close” male social networks that existed in study communities, which typically included four to ten men. To evaluate the intervention, we needed treatment and control groups that were as independent as possible, so we sought assignments of high-degree nodes to promoters with matched controls that minimised contamination across the treatment and control groups. This is non-trivial, as many of the peers of popular leaders are themselves popular, therefore connected to others widely throughout the beach, which would naturally create cross-contamination. Thus, to identify peer leadership clusters with minimal connections between treatment and control groups, we followed the graph cluster randomisation approach.^18^

Our search algorithm proceeded in two phases. The first phase enumerated a fixed number of promotor-led clusters with matching control cases, while the second phase then sorted all the first-phase solutions to find the one with the minimum number of social contacts between control and treatment groups. The first phase proceeded by randomly sampling a node with higher-than-average degree centrality and assigning them and all their peers to a treatment cluster: we first limited the network to multiplex (≥2 types of ties) relations. We then randomly selected treatment cluster leaders (promoters) with probability proportion to number of contacts (degree) and added the promoter and their direct contacts to the treatment group. We next randomly sampled a control promoter who had a degree matching our treatment case and assigned them and all of their direct contacts to the control group, ensuring balance across groups. We then repeated this process with all unassigned nodes, and the first phase was finished when the target N of clusters in each setting was met. This phase was repeated 1000 times. For phase 2, we selected the phase 1 assignment with the lowest overlap between control and treatment groups.

The social network data were then visualised, and metrics of social cohesion and embeddedness generated for each of the fishermen’s male social networks in each of the communities. Structural cohesion is a property of social networks defined by Moody and White^19^ as the extent to which multiple independent chains of relationships (relational pathways) among all pairs of individuals (actors, or nodes) within a network can act to hold it together: a group’s structural cohesion is equal to the minimum number of actors, *k*, who, if removed from the group, would disconnect the group (this is equivalent to the minimum number of independent paths linking each pair in the group). Social cohesion is an empirically based measure of diffusion potential (of information, resources, or normative influence) that varies within a network, as people are strongly linked to some but not to all people in the community. To assess overall community cohesion, we averaged the highest level of *k*-cohesion that each pair of actors jointly participates in, which is interpreted as the number of independent relational pathways connecting average pairs in the network, to assess the diffusion potential of the network. Finally, we present measures of relational tie strength (distribution of different relation types that men have with each other), distributions of embeddedness, and average pairwise connectivity along with sociograms of each community’s male social network.

### Role of the funding source

The funder of the study had no role in study design, data collection, data analysis, data interpretation, or writing of the manuscript.

## Results

**Figure 2:**
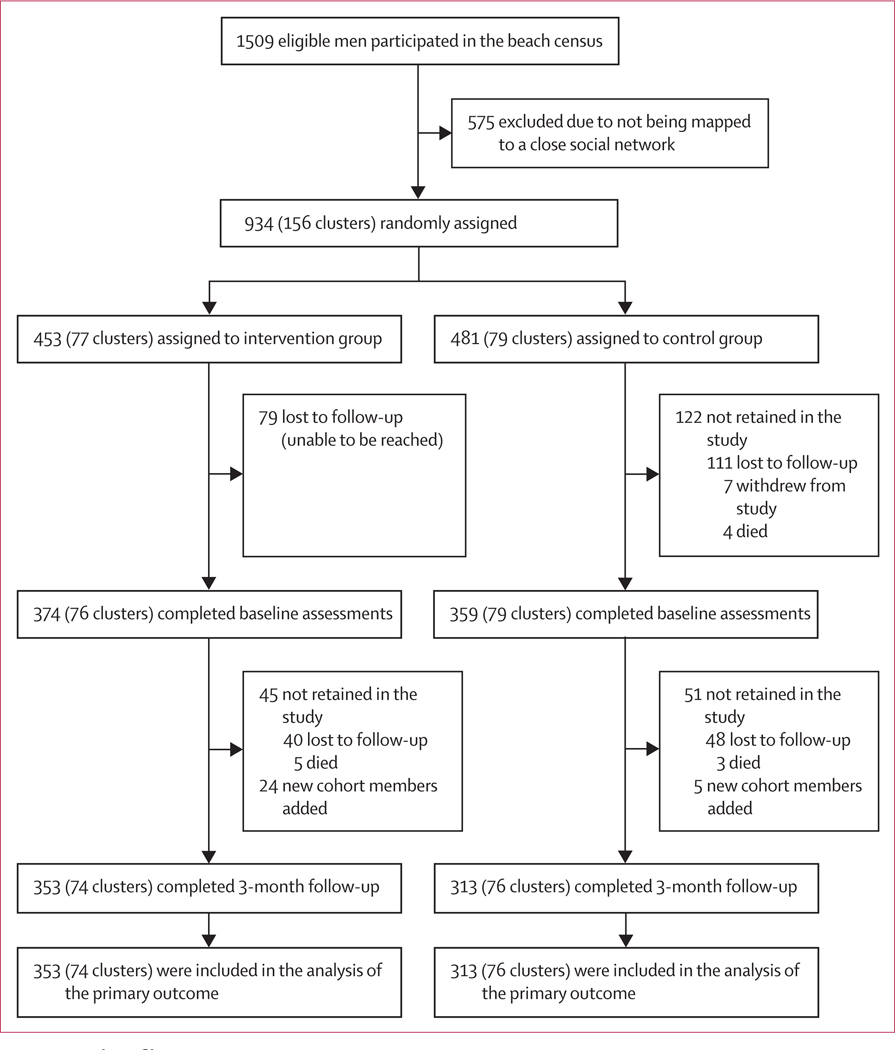
Trial profile

Between July 17, 2020 and Feb 2, 2022, we enumerated 1509 fishermen in three beach communities. Among these fishermen, 934 were mapped to social networks and comprised the intention-to-treat sample, after 575 were excluded due to not being mapped to a close social network. 453 men were randomly assigned to the intervention group and 481 were randomly assigned to the control group. 733 men were reached and completed a baseline survey (374 in the intervention group and 359 in the control group). 353 men in the intervention group and 313 in the control group completed the 3-month follow-up assessments and were included in the analysis of the primary outcome (figure 2).

Participants’ median age was 35·5 years (IQR 30·1–42·3); 626 (85%) of the 733 respondents were married and among those married, 138 (22%) were in polygynous marriages (table 1). Approximately two-thirds (68%) had completed primary education or below, and food insecurity in the past month was highly prevalent (92%). Reported “fair” or “poor” health status was at 42%, and hazardous alcohol consumption was at 17%. Levels of sexual partnership concurrency (overlapping sexual partnerships within any given month) were prevalent at 40% in the previous 6 months, and 12% of men reported having had a higher-risk sexual partner (reported as casual, commercial sex worker, one night stand, inherited partner, or stranger) in the same period. Condom use was rare, with 95% of 714 sexually active men reporting that they had not used a condom with any sexual partner in the previous 6 months. Self-reported current use of oral PrEP was also rare (48 [7%]).

**Figure 3:**
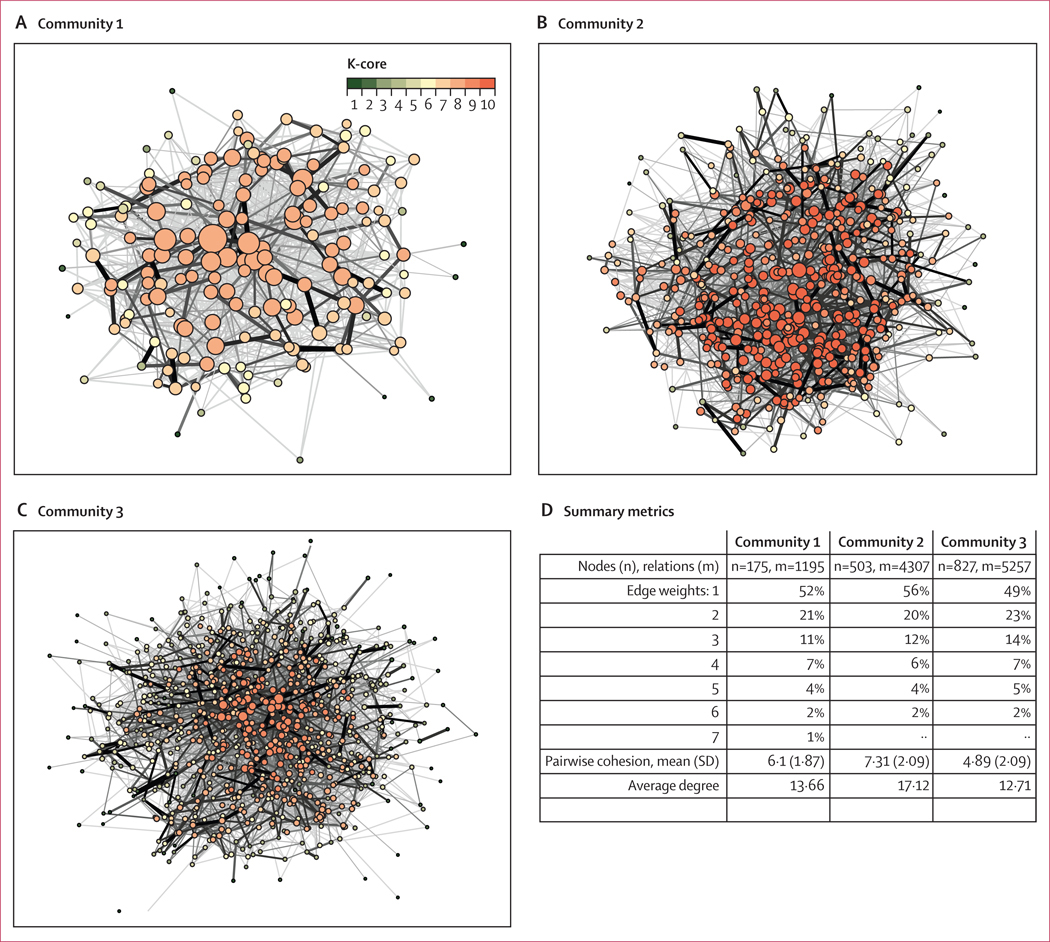
Male social networks of fishermen in beach communities, Siaya County, Kenya Spatial representations of male social networks of fishermen in each of the three study communities (A–C) and summary metrics (D). Each circle is a fisherman (node), and the size of the circle is proportional to the number of ties. N=number of nodes, and m=number of (undirected) links. Line thickness is proportional to the number of relations for each tie. The K-core scale displays the distribution of embeddedness of nodes in the network by colour, from lowest number of ties (n=1) shaded dark green to highest number (n=10) shaded red.

Sociograms of the male social networks of fishermen in the three study communities, along with measures of structural cohesion and embeddedness, are shown in figure 3. The social network of the first community was small, with n=175 nodes and m=1195 ties. The network was broadly cohesive, where most nodes were connected to other nodes throughout the whole network, forming a single major core with only a small periphery. The edge weight distribution shows that most ties (52%) were single relation, meaning that these men were socially connected to each other through a single relation type (eg, work, sharing health information, or financial matters) However, about 15% of the nodes shared four different types of relations, indicating strong relationships for cases in which men had multiple domains of communication, and these were approximately evenly distributed throughout the network.

The second network was larger, with n=503 nodes and m=4307 ties. The overall structure of the network was similar to the first network, but with a larger single cohesive core and an even smaller periphery. As in the first community, most ties were unidimensional: 56% were based on a single relational type. However, 12% shared four different types of relations. The third network was the largest, with n=827 nodes and m=5257 ties. A basic unified shape was again seen in this network, but the distribution of the cohesion structure differed somewhat from the others. As in the other communities, weak ties comprised the largest set of ties, with about 49% based on a single relation type, although a small proportion of nodes had deep, strong ties with other men in the network. Combined, we mapped 156 clusters of an average size of 5·98 (coefficient of variation, 0·7).

We administered follow-up surveys to 666 men at 3 months, of whom 496 (74·5%) were baseline cluster members, 141 (21·2%) were baseline promoters, and 29 (4·4%) were recruited by promoters as new cluster members but were not part of the original mapping. From the original intention-to-treat sample (n=934), 270 were coded as failure (ie, not testing) due to missing data; 201 of these were among those who were never enrolled in the study despite being mapped. Participants known to be living with HIV for more than 3 months via confirmed medical records or who died were excluded (n=164 and n=12, respectively; appendix). One participant reporting testing and seroconverting during the 3-month follow-up period and was not coded as missing. Approximately half of the intention-to-treat sample (48·1%) self-reported HIV testing via any modality in the past 3 months. In the intention-to-treat sample, the cluster-adjusted proportion of HIV self-testing via any modality was higher among men in the intervention group (65·6%) than in the control group (31·3%; overall p<0·0001; table 2). The cluster-adjusted proportion of HIV testing with a self-test was higher among men in the intervention group (60·4%) than in the control group (10·0% overall p<0·0001). In multilevel models with the intention-to-treat sample, the intervention was associated with 2·06 times the odds of recent testing (95% CI 1·68–2·54; p<0·0001) and 6·04 times higher odds of recent testing with a self-test (4·33–8·42; p<0·0001) compared with the control group.

Regarding linkage to facilities and uptake of HIV prevention (oral PrEP or ART), the cluster-adjusted proportion of linkage to a study-affiliated health-care facility for confirmatory testing was 67·3% among men in the intervention group and 15·6% (overall p<0·0001) in the control group among those who tested. In multilevel models with the intention-to-treat sample, the intervention was associated with a 4·32 times higher odds of linkage to a study-affiliated health facility (95% CI 3·18–5·86, p<0·0001) than the control group. Among those who linked to the health facility and screened as eligible, the cluster-adjusted proportions of oral PrEP uptake did not differ between the intervention (19·7%) and control group (19·6%; p=0·99), nor was the intervention associated with an increase in the adjusted odds of PrEP initiation (odds ratio 1·38 [95% CI 0·80–2·37]; p=0·24). Three individuals tested positive for HIV and initiated ART (table 2); the sample was insufficient for hypothesis testing.

Throughout the study follow-up period, 14 adverse events were reported among those enrolled in the study, including 13 deaths and one fracture. None of the adverse events were related to study participation.

## Discussion

Among the highly mobile population of fishermen in Kenya, this study shows that utilising men’s social networks, HIV self-tests, and small financial incentives can significantly increase HIV testing coverage and linkage to health facilities. In social networks in which a network-central man was given multiple HIV self-tests to promote testing in their networks as well as transport vouchers to incentivise linkage to prevention or treatment, HIV testing was much higher than in comparison clusters where men had to visit existing health facilities to get tested. The intervention clusters also had significantly higher rates of linkage to health facilities for confirmatory testing and screening for PrEP eligibility for those newly diagnosed or needing re-engagement. The combination of social networks and small financial incentives can therefore facilitate engagement in HIV services among the hardest-to-reach people in settings with a high burden of HIV.

This study contributes to the growing literature on HIV self-testing and highlights optimal ways to use self-tests to increase HIV testing coverage in sub-Saharan Africa. Although self-tests are highly acceptable among men as well as in key and priority populations in which testing coverage remains suboptimal, identifying the best way to get self-tests in the hands of people who need them the most remains a challenge in many countries. Although previous studies have demonstrated the promise of secondary distribution of self-tests by women to their male partners,^13–15^ this is one of the first studies to show that distribution of self-tests by network-central men can also reach a high proportion of men. Nearly two of every three fishermen in social networks obtained an HIV test because of the social network-based approach to distributing self-tests. Our findings are consistent with a pilot study of the secondary distribution approach in peer networks in fishing communities in Uganda, although that study was a single-arm trial.^20^ Relying on the social networks of highly connected men can thus be very useful for increasing testing coverage among hard-to-reach men.

Because HIV self-tests offer users the ability to privately learn their HIV status, measuring and facilitating linkage to treatment and prevention after self-testing has proven to be a major challenge. As with other community-based testing modalities including home-based testing, linkage after HIV self-testing is often suboptimal without other interventions to motivate confirmatory testing and linkage to care and prevention. A recent WHO summary of HIV self-test studies reported that post-HIV self-test linkage to clinics was 50–56% in general populations in sub-Saharan Africa.^21^ We found that small financial incentives coupled with the support from promoters resulted in linkage to care and prevention that was more than four times higher than in the control group, and that nearly 70% of men in the intervention group sought facility-based confirmatory testing and screening for prevention or treatment after self-testing. Targeted use of small financial incentives might therefore be highly effective in maximising the benefits of HIV self-testing.

Our findings are consistent with previous research showing that financial and non-financial incentives have been effective in promoting utilisation of HIV services in low-income and middle-income countries.^16^ They have also been utilised by governments and non-governmental organisations to promote utilisation of some health services such as antenatal care, facility deliveries, and childhood vaccinations.^22^ Although we did not separately evaluate the effectiveness of provision of financial incentives only, the results suggest that small incentives have the potential to result in increased linkage to treatment and prevention services among men who have used an HIV self-test. Additional studies, including cost-effectiveness analyses and implementation research, are necessary to determine whether to scale up the use of incentives within the context of self-testing interventions.

In addition to the financial incentives, it is worth noting that the network-central men (ie, the promoters) might also have played a role in encouraging linkage to facilities. However, since the promoters in the control group were also asked to encourage linkage to HIV services, it is unlikely that the differences in post-test linkage between intervention and control groups were driven entirely by the presence of the promoters. Beyond incentives and promoters, the use of self-tests might also have motivated users to link to care.

Almost 20% of men who linked to a health facility initiated PrEP, but this did not significantly differ by study group. However, because the intervention increased HIV testing and linkage to a health facility, overall PrEP uptake was much higher in the intervention group than the control group. However, additional interventions are likely to be necessary for promoting oral PrEP uptake. Furthermore, the relative unpopularity of oral PrEP could limit the levels of uptake that could be achieved. Although it is highly effective for preventing HIV infection, sustained use of oral PrEP remains consistently low, greatly hindering its epidemic control potential.^23,24^ The barriers to oral PrEP use might outweigh its benefits for many potential users in sub-Saharan Africa. These include stigma due to the association of PrEP use with HIV risk behaviours, perceptions that oral PrEP medications are indistinguishable from ART treatment medications, side-effects and pill burden, unsupportive partners, and difficulties with care engagement due to mobility.^25,26^ From a public health perspective, PrEP uptake among 20% of HIV seronegative fishermen who linked to facilities could be considered a success. Other studies in the region have achieved similar findings only after employing extensive health system supports to promote PrEP uptake: for example, the SEARCH trial in Kenya and Uganda, which offered out-of-facility options for PrEP initiation, and extensive programmatic support including PrEP follow-up visits at facilities, homes, and community locations, found PrEP initiation within 90 days of HIV testing was 27% among adults at elevated risk.^27^ Data on PrEP uptake and current use among men in general populations in Africa (not cohorts of men who have sex with men) remain rare: in a Ugandan fishing community, 18·9% of men had ever used PrEP and nearly half of those men had discontinued at the time of the survey;^28^ in Kenya, 15% of adults initiating PrEP are in the general population.^29^ The biomedical HIV prevention toolkit is expanding to include more user-friendly prevention products such as injectable long-acting cabotegravir and long-acting lenacapavir, both of which show promise for addressing the challenges to oral PrEP use.^29^ This study’s social network-based approach has proved to facilitate men’s ease of entry to health facilities, but newer prevention modalities might be needed to facilitate prevention uptake.

In this study, the fishermen’s social networks were highly cohesive, particularly compared with previously studied rural networks.^30,31^ The social organisation of fishing work—with men working together on different boats and travelling between different sites—probably promotes fluid and expansive relations.^32^ Although our intervention tested the direct effects of promoters on their immediate peers, the structure of these networks is likely to be conducive to easy information diffusion, so there might well be further unmeasured spillover between cluster members and their non-cluster peers. In settings with this sort of easy diffusion potential, information about treatment and care can readily spread through the community. This represents a clear opportunity to promote health behaviour via trusted peers which can be an effective and efficient way to reach many people and might contribute to generalised health across multiple domains. Conversely, much care is needed to ensure that attempts to leverage networks for health interactions are respectful and community-promoting, as negative experiences are also likely to be shared, which could spread quickly and damage health-promotion activity. For example, if oral PrEP was deemed unpopular among socially influential men, this might increase hesitancy of other men in the network to enrol in PrEP.

This study has several limitations. First, the primary outcome of HIV testing uptake was self-reported by participants. Measurement of actual behaviour is a challenge in nearly all HIV self-testing studies and programmes. Although we assessed HIV testing behaviours with self-reports in both study groups, it remains a possibility that participants in the intervention group were more likely to over-report HIV testing behaviour since they were likely to have received self-tests from promoters in their network. However, since clinic-based data show increased PrEP counselling in the intervention group compared with the control group, it is unlikely that the differences in testing uptake between groups were exaggerated by self-reports. Moreover, baseline data showed low levels of self-reported use of HIV self-tests across both groups, but low self-test use only in the control group at 3-month follow-up; in addition, use of self-tests was validated by the return of used tests to the facilities in the intervention group. Second, a methodological limitation inherent to our study design is that the intention-to-treat sample includes individuals for whom we have no data and with whom our study did not interact. Network treatments commonly generate so-called spillover to people who are connected to those treated but not treated themselves,^33^ which is not captured by our research design, as such cases are not sampled. It is likely that our intervention affected these cases in some way, although we cannot know what the effect size would have been. Our intervention was also subject to implementation challenges: while we intended in the control group for referral vouchers to be redeemable at health facilities for an HIV self-test, shortages at facilities meant that only facility-based testing was available during the study period. Finally, implementing the intervention required conducting social network mapping to identify network-central individuals who were then recruited to receive the self-tests and offer them to their network members. A high-scale implementation of this approach is likely to be hindered by the cost and complexity of such mapping, thus limiting the effect of the intervention. However, recent research demonstrates there are simpler, lower-cost ways to identify network-central individuals, which have the potential to greatly enhance the feasibility and effect of the intervention.^34^

The influence of social networks on health behaviours and outcomes is well established, and network interventions use this influence purposefully to accelerate behaviour change and achieve positive health outcomes. The findings from this study demonstrate that when coupled with a social network-based approach, HIV self-tests and financial incentives can be highly effective in promoting better health outcomes among men in sub-Saharan Africa. Our intervention is scalable and could have a major effect on testing and treatment outcomes in the region.

## Supplementary Material

Supplementary Appendix

## Figures and Tables

**Table 1:** Baseline characteristics

	Intervention group (n=374)	Control group (n=359)	Total (n=733)

Median age, years (IQR)	34·5 (28·9–41·2)	37·2 (31·9–43·4)	35·5 (30·1–42·3)
Marital status			
Married	320 (86%)	306 (85%)	626 (85%)
Unmarried but partnered	15 (4%)	15 (4%)	30 (4%)
Single, divorced, or widowed	39 (10%)	38 (11%)	77 (11%)
Education			
Primary school or less	259/373 (69%)	241/358 (67%)	500/731 (68%)
Some or all of secondary school	93/373 (25%)	97/358 (27%)	190/731 (26%)
Secondary school and further	21/373 (6%)	20/358 (6%)	41/731 (6%)
Polygynous marriage*	66/320 (21%)	72/306 (24%)	138 (22%)
Fishing is primary source of income	310 (83%)	312 (87%)	622 (85%)
Hazardous drinking†	61 (16%)	62 (17%)	123 (17%)
Food insecure	348 (93%)	330 (92%)	678 (92%)
Health status			
Very good	61/372 (16%)	48/357 (13%)	109/729 (15%)
Good	166/372 (45%)	151/357 (42%)	317/729 (43%)
Fair	126/372 (34%)	143/357 (40%)	269/729 (37%)
Poor	19/372 (5%)	15/357 (4%)	34/729 (5%)
Sexual risk behaviour over the past 6 months			
Any concurrent sexual partner‡	143/356 (40%)	136/337 (40%)	279/694 (40%)
Any high-risk sexual partner§	36/366 (10%)	50/348 (14%)	86/714 (12%)
No condom use with any partner	344/365 (94%)	335/348 (96%)	680/713 (95%)
Current PrEP use	30/374 (8%)	18/359 (5%)	48/733 (7%)
Ever tested for HIV	367/374 (98%)	345/359 (96%)	712/733 (97%)
Ever heard of HIV self-testing	273/374 (73%)	273/359 (76%)	546/733 (75%)

Data in parentheses are column percentages unless otherwise indicated. PrEP=pre-exposure prophylaxis.

* Asked of married men.

† Defined as an AUDIT-C score of 4 or greater.

‡ Two or more overlapping sexual partners within any given month over the past 6 months.

§ Sexual partner reported as casual, commercial sex worker, one night stand, inherited partner, or stranger within the last 6 months.

**Table 2:** HIV testing and linkage to care among fishermen in the *Owete* study

	Sample size	Intervention group* (%)	Control group* **(%)**	p value	Odds ratio (95% CI)†	p value	Relative risk (95% CI)†	p value

Any HIV testing within the past 3 months	768‡	65.6	31.3	<0.0001	4.29 (2.94–6.27)	<0.0001	2.06 (1.68–2.54)	<0.0001
HIV self-test use within the past 3 months	768	60.4	10.0	<0.0001	14.21 (9.25–21.81)	<0.0001	6.04 (4.33–8.42)§	<0.0001
Linkage to health facility	934¶	67.3	15.6	<0.0001	11.59 (7.45–18.03)	<0.0001	4.32 (3.18–5.86)§	<0.0001
PrEP uptake	314||	19.7	19.6	0.99	1.58 (0.75–3.31)	0.26	1.38 (0.80–2.37)	0.24
ART initiation**	3	..	..	..	..	..	..	..

ART=antiretroviral therapy. PrEP=pre-exposure prophylaxis.

* Cluster-adjusted mean.

† Models adjusted for beach community and used cluster-robust standard errors to control for cluster.

‡ Any HIV testing with the past 3 months is reported for those in the intention-to-treat sample who were not known to be HIV infected at study entry (n=768).

§ Modelled with a modified Poisson (with log link and Poisson family) given failure to converge with generalised linear model.

¶ Sample is intention to treat (n=934).

|| PrEP uptake is reported for those who linked to care and were not known to be HIV infected (n=314).

** Insufficient sample size for hypothesis testing.

## Data Availability

De-identified study data and a data dictionary will be made available in a secure online repository approximately 1 year after completion of the ongoing trial (NCT04772469) following Owete Scientific Committee approval of a concept sheet summarising the analyses to be done, with a signed data access agreement. Further inquiries can be directed to the Owete Scientific Committee via carol.camlin@ucsf.edu.

## Research in context

### Evidence before this study

Despite significant gains in men’s engagement in HIV testing, prevention, and treatment in many countries in sub-Saharan Africa, current engagement rates remain suboptimal. Research has identified mobile men, particularly those involved in the fishing and truck driving trades, as a subgroup of men at highest risk of HIV infection with low rates of HIV testing. With the advent of HIV self-testing and the success of social network-based peer interventions among men, opportunities exist to test the efficacy of a combined intervention to increase mobile men’s engagement with HIV testing and linkage to HIV prevention and treatment. We searched PubMed for previous studies of HIV self-testing or social network interventions among men in Africa using the terms (“HIV self-testing” OR “social network intervention” AND “men” AND “Africa”) on Feb 27, 2024, with no publication date or language restrictions, along with reviewing our pre-trial grant reference library for evidence justifying the need for the tested intervention. Of the 115 results from the PubMed search, most of the early evidence focused on the feasibility and acceptability of HIV self-testing with a predominance of qualitative approaches over survey data and randomised comparisons, all indicating the feasibility and desirability of HIV self-testing for men including subgroups of men at high risk and male partners of pregnant women and female sex workers. We identified 15 randomised controlled trials of HIV self-testing for men directly or as secondary distribution that uniformly showed significant effects in increasing HIV testing for men. Evidence for the potential of social network interventions for men in Africa was found in four studies, with three specifically addressing HIV testing or self-testing. Several previous studies have addressed the preferences for HIV self-testing among mobile truck drivers and fishermen. We found no randomised trials of a combination social network and HIV self-testing intervention approach for mobile men.

### Added value of this study

This study presents positive findings from a randomised controlled trial of a combined social network and HIV self-testing intervention to improve HIV testing and linkage to HIV prevention and treatment for mobile men at high risk for HIV and its consequences. Providing multiple self-tests to men and encouraging them to distribute tests to other men in their social networks (ie, secondary distribution) has been a promising but untested strategy to increase men’s testing; this is the first randomised controlled trial to assess the effect of secondary distribution of self-tests in men’s social networks. The study provides evidence that a social network-based approach, involving recruitment of network-central men to act as leaders and distributors of self-tests, is an optimal strategy for increasing HIV testing among high-risk individuals not reached by other HIV testing modalities, such highly mobile men. Additionally, providing a low-cost incentive was effective at motivating men’s confirmatory testing and linkage to care and prevention, showing that this additional intervention can maximise the HIV prevention potential of HIV self-testing.

### Implications of all the available evidence

The existing evidence on the feasibility and acceptability of HIV self-testing and the new findings of significant improvements in HIV testing and linkage to HIV prevention and care following a combined social network and HIV self-testing intervention provide a clear way forward on how to increase mobile men’s engagement in HIV testing and care. Implications for further research include assessing the efficacy of the combined approach in other high HIV risk populations and developing approaches for cost-effective scale-up of the intervention.
