# Supplementary material for: The effect of a social network-based intervention to promote HIV testing and linkage to HIV services among fishermen in Kenya: a cluster-randomised trial
Source: Lancet Glob Health. Author manuscript; Available in PMC 2025 Sep 12. (PMC12431777; doi:10.1016/S2214-109X(24)00539-4)
Supplement: Supplementary Appendix [file NIHMS2099423-supplement-Supplementary_Appendix.pdf]

THE LANCET
Global Health

Supplementary appendix

This appendix formed part of the original submission and has been peer reviewed. We post it as supplied by the authors.

Supplement to: Camlin CS, Sheira LA, Kwena ZA, et al. The effect of a social network-based intervention to promote HIV testing and linkage to HIV services among fishermen in Kenya: a cluster-randomised trial. *Lancet Glob Health* 2025; **13:** e669–78.

Supplementary Table 3: Treatment Status of *Owete* Participants Confirmed via Medical Records to be Living with HIV (n=164)

	Intervention	Control	Total
**Living with HIV**	79 (48·2)	85 (51·8)	164
**Years living with HIV (median, IQR)**	6·4 (3·8, 11·5)	8·0 (4·7, 11·0)	7·6 (4·1, 11·2)
**Baseline HIV-1 RNA viral load, copies/mL (median, IQR)**	50 (0, 323)	50 (0, 250)	50 (0, 268)
**WHO Stage at enrolment**			
1	36 (49·3)	38 (51·4)	74 (50·3)
2	22 (30·1)	22 (29·7)	44 (29·9)
3	12 (16·4)	14 (18·9)	26 (17·7)
4	3 (4·1)	0 (0)	3 (2·0)
**Most recent HIV-1 RNA viral load, copies/mL (median, IQR)**	21 (0, 85)	0 (0, 99)	5 (0, 88)

Supplementary Table 4 Adjusted associations between HIV Testing and Linkage to Care among Fishermen in the *Owete* Study

	**Odds ratio 95% CI[1]**	**p-value**	**Relative risk 95% CI[2]**	**p-value**
**Any HIV testing ≤3 months**	4·01 (2·55, 6·28)	<0·0001	1·70 (1·42, 2·03)	<0·0001
**HIV self-test use ≤3 months**	14·07 (8·88, 29·30)	<0·0001	4·73 (3·44, 6·53)	<0·0001
**Linkage to health facility**	12·14 (7·48, 19·69)	<0·0001	3·73 (2·76, 5·02)	<0·0001
**PrEP uptake**	1·45 (0·65, 3·28)	0·36	1·27 (0·73, 2·20)	0·39
**ART initiation[3]**	--	--	--	--

[1] Models adjusted for community and age and had cluster-robust standard errors to control for cluster.
[2] Modelled with a modified Poisson (with log link and Poisson family) given failure to converge with generalized linear model
[3] Insufficient sample size for hypothesis testing
